# Merkel cell carcinoma: epidemiology, clinical features, diagnosis and treatment of a rare disease^☆^

**DOI:** 10.1016/j.abd.2022.09.003

**Published:** 2023-03-02

**Authors:** Stella Oliveira Meireles Siqueira, Gabriella Campos-do-Carmo, Alexssandra Lima Siqueira dos Santos, Cícero Martins, Andreia Cristina de Melo

**Affiliations:** a Division of Clinical Research and Technological Development, Instituto Nacional de Câncer, Rio de Janeiro, RJ, Brazil; b Gávea Medical Center, Rio de Janeiro, RJ, Brazil; c Section of Clinical Oncology, Instituto Nacional de Câncer, Rio de Janeiro, RJ, Brazil

**Keywords:** Carcinoma, Merkel cell, Immune checkpoint inhibitors, Merkel cell polyomavirus, Skin neoplasms

## Abstract

Merkel cell carcinoma is a rare skin cancer with neuroendocrine differentiation. The risk factors include sun exposure, advanced age, immunosuppression (such as transplant recipients, patients with lymphoproliferative neoplasms, or patients with HIV), and Merkel cell polyomavirus infection. Clinically, Merkel cell carcinoma appears as a cutaneous or subcutaneous plaque or nodule, but this tumor diagnosis is rarely made clinically. Therefore, histopathology and immunohistochemistry are usually necessary. Primary tumors without evidence of metastases are treated with complete surgical excision and appropriate surgical margins. The presence of occult metastasis in a lymph node is frequent and a sentinel lymph node biopsy should be performed. Postoperative adjuvant radiotherapy increases local tumor control. Recently, agents that block the PD-1/PD-L1 pathway have shown objective and durable tumor regression in patients with advanced solid malignancies. The first anti-PD-L1 antibody used in patients with Merkel cell carcinoma was avelumab, but pembrolizumab and nivolumab have also shown efficacy. This article describes the current state of knowledge of the epidemiology, diagnosis, and staging of Merkel cell carcinoma, as well as new strategies for its systemic treatment.

## Introduction

Merkel Cell Carcinoma (MCC) is an unusual and aggressive type of skin cancer with neuroendocrine origin,^1^ and was first described in 1972 by Toker as a trabecular skin carcinoma.^2^ Since then, understanding of its pathophysiology, outcomes and management have improved, especially after immunotherapies. Also known as primary cutaneous neuroendocrine carcinoma, MCC shares features with Merkel cells of the skin, justifying its nomenclature.^3^

## Epidemiology

The incidence rate of MCC varies in different regions of the world. In the European Union, between 1995 and 2002, its annual incidence rate was 0.13 per 100,000 inhabitants, but higher in groups aged 65 years or older.^4^ In the United States, the incidence rate was 0.79 per 100,000 inhabitants in 2011.^5^ In 2013, Paulson et al. reported that the absolute number of MCC in the United States was 2488 cases, which corresponds to an incidence rate similar to 0.7 per 100,000 person-years.^6^

A higher incidence of MCC was observed in an Australian study, with a rate of 1.6 per 100,000 inhabitants in the state of Queensland between 2006 and 2010, more frequent in males (2.5 per 100,000) than in females (0.9 per 100,000) and with an average life expectancy of 75.5 years for men and 78 years for women at the diagnosis.^7^

Although rare, the incidence of MCC has increased dramatically.^8^ In 2018, Paulson et al. reported that the number of cases increased 95% between 2000 and 2013 in the US, compared with an increase of 57% for melanoma and 15% for all other solid tumors.^6^ In Australia, the overall incidence of MCC increased on average by 2.6% per year between 1993 and 2010 (95% CI: 1.1%‒4.2%).^7^ MCC is often underdiagnosed and part of its increased incidence is thought to be due to better-trained pathologists and also to the development of biomarkers that have improved disease detection.^6^

Regarding the epidemiology of MCC in Brazil, Melo et al. published a study with data collected from Population-Based (2000–2015) and Hospital-Based (2000–2017) Cancer Registries. A total of 881 patients with the disease were analyzed, most of which were female (51.2%), aged over 60 years (82.2%), white (67.6%), and diagnosed predominantly in stages III or IV (50.5%). Moreover, age-standardized mean incidence rates were found to increase significantly in men between the years 2000 (0.31/1,000,000) and 2015 (1.21/1,000,000), with an annual percentage variation of 9.4 (95% CI: 4.7–14.4; p < 0.001). Meanwhile, in women, incidence rates did not increase significantly over the period.^9^

Carneiro et al. retrospectively evaluated 32 patients treated at the National Cancer Institute with MCC between 2002 and 2012, and the mean age at diagnosis was 72 years. However, unlike the international data, most patients were female (69%).^10^

## Risk factors

Several studies suggest that multiple factors may contribute to the development of MCC.^7^, ^8^, ^11^, ^12^, ^13^ Sun exposure is an important risk factor,^13^, ^14^ supported by the higher incidence of MCC in regions with higher rates of ultraviolet radiation,^7^, ^8^ and the greater tendency to occur in sun-exposed areas, such as the head and neck region.^15^ Lunder et al. also showed a higher occurrence of MCC in patients with a history of psoralen use and skin exposure to longwave ultraviolet radiation.^16^ MCC is also more frequent in Caucasian patients compared to other ethnicities.^8^

Immunosuppression is also a risk factor,^13^ therefore, MCC is more common in transplant recipients, patients diagnosed with lymphoproliferative malignancies (such as chronic lymphocytic leukemia), or patients with HIV.^8^, ^14^

In addition, advanced age is also considered a risk factor.^13^, ^15^ In a US series, 90% of all patients with MCC were aged over 50 years, 76% were over 65 years, and 49% were over 75 years.^8^, ^15^

In 2008, Feng et al. first described the association between a novel polyomavirus and MCC, identifying viral DNA in 8 of 10 Merkel tumors, suggesting that viral infection could be an early event in the pathogenesis.^11^

## Polyomavirus associated with Merkel cell carcinoma

The discovery of the MCC-associated polyomavirus in 2008 revolutionized the knowledge about tumor pathogenesis, and it was named Merkel cell polyomavirus (MCPyV).^11^ The association was identified using a digital transcriptome subtraction technique, which is a bioinformatics method used to detect the presence of novel pathogen transcripts through the computational removal of host sequences; therefore, in this case, known human sequences were filtered to identify potential viral transcripts.^3^

Cases of subclinical MCPyV infection increase with senescence, reaching a prevalence of 60% to 80% in adults. However, this may differ significantly in other geographic regions, such as Australia, where a much lower association with viral infection has been reported, of around 25%.^17^ The skin constitutes the major viral infection site, although the virus has also been detected in peripheral blood and other organs. MCPyV infection seems to be asymptomatic.^3^

The *Polyomaviridae* family, to which MCPyV belongs, consists of small double-stranded DNA viruses. It includes other polyomaviruses associated with cutaneous infection in humans (*Trichodysplasia spinulosa* polyomavirus, human polyomavirus 6, and human polyomavirus 7) or disease in other organs and systems (such as JC virus, a ubiquitous human polyomavirus that causes central nervous system disease in immunocompromised patients, including progressive multifocal leukoencephalopathy, granular cell neuropathy, and JC virus encephalopathy). To date, MCPyV is the only human oncovirus in the *Polyomaviridae* family, but the reason for this distinct status is not yet known.^3^

The specific type of host cell for infection by this polyomavirus remains unclear. Merkel cells are thought to be insufficiently numerous to explain the viral load that is normally detected in the skin, and monocytes in peripheral blood have been implicated as possible reservoirs of infected cells.^3^

The episomal viral genome has both early and late regions. The former have genes that encode proteins responsible for coordinating viral replication, whereas the latter regions are linked to viral capsid proteins. It seems that the integration of MCPyV DNA into the host genome occurs shortly after the infection.^3^, ^18^

**Figure 1:**
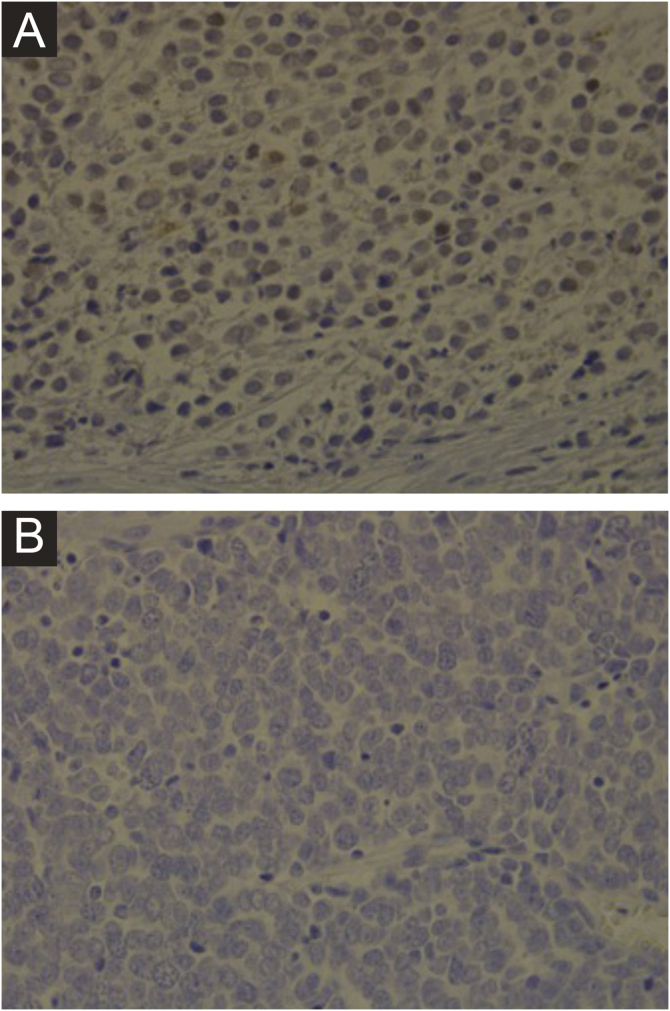
Immunohistochemistry for MCPyV performed on Merkel cell carcinoma slides. (A) Immunohistochemistry slide from a patient showing a positive result for MCPyV (×400 magnification). (B) Immunohistochemistry slide from a patient showing a negative result for MCPyV (×400 magnification).

Immunohistochemistry, PCR, *in situ* DNA hybridization, or next-generation sequencing technologies are available methods to detect MCPyV in tumors, but these tests vary greatly in sensitivity and specificity. The development of a monoclonal antibody used for the specific detection of the large T (LT) antigen through immunohistochemistry ‒ exclusive for MCPyV (the CM2B4 clone) ‒ allowed the detection and observation of MCPyV *in situ*. This method is commercially available and has approximately 88% sensitivity and 94% specificity.^3^ Fig. 1 shows examples of positive and negative immunohistochemistry results for Merkel cell polyomavirus performed using the anti-MCPyV large T-antigen (CM2B4) antibody.

## Clinical features

**Figure 2:**
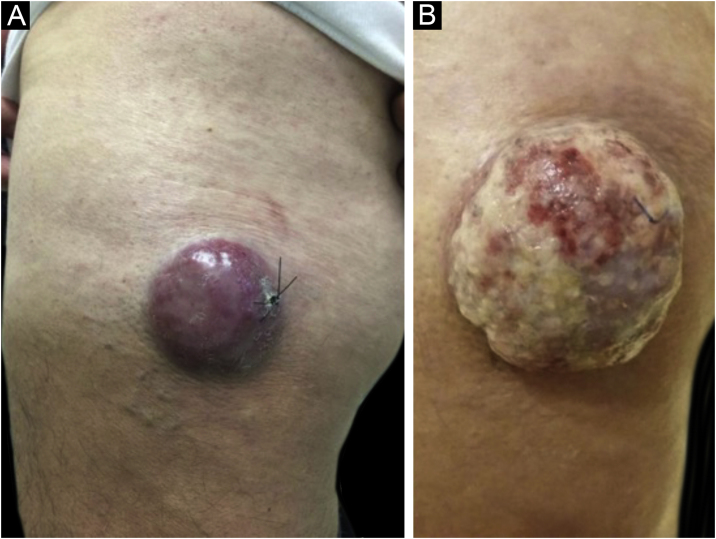
Merkel cell carcinoma located on the left thigh of a male patient. (A) At diagnosis, tumor lesion with cupuliform appearance, with erythema and mild desquamation. (B) Tumor evolution with growth and ulceration throughout the lesion. Source: Courtesy of Dr. Gabriella Campos-do-Carmo (the patient consented to the use of images for educational purposes).

Clinically, MCC can present as a cutaneous or subcutaneous nodule, and even have a cystic appearance. The color can vary between red, pink, blue, violet, or skin color. Initially, the lesions are usually painless and solitary, but they may also ulcerate or be surrounded by satellite lesions. At diagnosis, the dimensions can vary in size but are usually smaller than 20 mm, and most cases show rapid tumor growth in a few months^15^ (Fig. 2).

The lesions usually appear on photoexposed areas, while 19% appear on the buttocks or areas minimally exposed to the sun. The most frequent primary anatomic sites are the head and neck (29%), followed by the lower limbs (24%) and the upper limbs (21%).^15^

Heath et al., in 2008, proposed the mnemonic rule “AEIOU” (Asymptomatic/lack of tenderness, Expanding rapidly, Immune suppression, Older than 50 years and Ultraviolet exposed site) based on the analysis of 195 cases, in an attempt to aid the diagnosis. In this cohort, 89% of the patients diagnosed with MCC met three or more criteria, 52% of the patients met four or more criteria, and 7% of the patients met all five criteria.^15^

At the time of diagnosis, up to 37% of the patients present with nodal disease and 6%–12% present with metastatic disease.^19^ About 15% of the patients have a affected lymph node without an identifiable skin lesion, probably reflecting regression of the primary site.^3^, ^15^

Distant metastases appear most often in non-regional lymph nodes, skin, bones, lungs and pleura or liver and, less commonly, in the pancreas, adrenal glands, brain, kidneys, subcutaneous tissue or muscle. Rare sites of metastases include breasts, the gastrointestinal tract, testes, heart, retroperitoneum, and the peritoneum.^14^

## Diagnosis

Due to the lack of specific features and symptoms, the diagnosis becomes a challenge. Therefore, the histopathological examination by an experienced pathologist and the staining with specific immunohistochemical markers are essential in this process.^3^

The North American organization National Comprehensive Cancer Network (NCCN) has proposed specific algorithms for the diagnosis of MCC, which include the following strategies: 1) A complete examination of the skin and lymph nodes; 2) Biopsy for histopathological and immunohistochemical analysis; 3) Sentinel lymph node biopsy (SLNB) for patients without clinically positive lymph nodes, preceding the excision, if possible; 4) Fine needle aspiration or lymph node biopsy for patients with clinically positive lymph nodes; consider open biopsy; 5) Imaging exams for staging as clinically indicated.^14^

Dermoscopy is a complementary exam that can be useful in some cases, but there is still a lack of more complete dermoscopic descriptions in the literature. MCC often exhibits a variety of dermoscopic vascular patterns, most commonly milky red areas/ globules, polymorphic vessels, and irregular linear vessels,^20^ reflecting rapid tumor growth.

**Figure 3:**
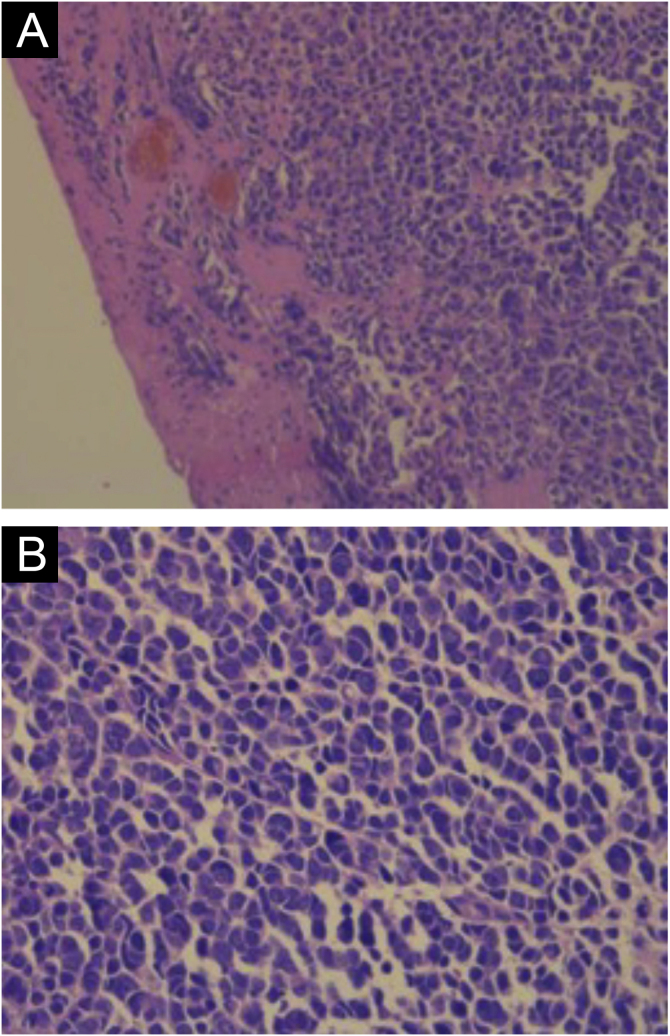
Histopathology of Merkel cell carcinoma with hematoxylin-eosin staining. (A) Cords of tumor cells in the dermis (×200 magnification). (B) Tumor cells showing scarce cytoplasm and round or irregular nuclei (×400 magnification).

Hematoxylin-eosin staining reveals the proliferation of small basophilic tumor cells in the dermis and/or hypodermis,^21^ with sparse cytoplasm, abundant mitoses, and dense cytoplasmic granules. Necrotic cells are also common.^8^, ^20^ Histopathology examples are depicted in Fig. 3.

Cytokeratin 20 (CK20) and neuroendocrine markers such as chromogranin A, synaptophysin, CD56, neuron-specific enolase, and neurofilaments are expressed in MCC.^3^, ^14^, ^21^ Paranuclear dot-like immunopositivity for CK20 is highly suggestive, whereas the neuroendocrine markers are non-specific.^3^

For patients with clinically negative nodes, SLND should be performed and its positivity rate ranges from 30% to 38%.^14^ When negative, a lower risk of recurrence,^22^ better disease free survival (DFS) and overall survival (OS) are observed.^14^

The use of imaging exams for the diagnostic investigation of patients with MCC is still under discussion. The NCCN does not recommend imaging exams in patients without clinical suspicion of affected lymph nodes. SLND is considered the most reliable test to identify metastases in non-clinically suspicious lymph nodes. Brain magnetic resonance imaging (MRI), neck, chest, abdomen, and pelvis computed tomography scans or PET-CT are indicated for surgical planning or when unresectable tumors or distant metastases are suspected.^14^

Gupta et al. have shown that computed tomography has low sensitivity, around 20%, for detecting lymph node metastases and low specificity for distant metastases.^22^ Therefore, although computed tomography has been used as a screening tool for regional or distant metastases in MCC, data supporting its application are limited.^14^, ^22^

PET-CT has been extensively assessed in patients with MCC.^14^ Colgan et al. retrospectively evaluated 36 patients with MCC who underwent PET-CT scans prior to SLNB and observed 83% and 95% of sensitivity and specificity, respectively.^23^

## Differential diagnosis

Clinically, the differential diagnoses for MCC are benign skin lesions, such as acneiform cyst, lipoma, dermatofibroma or fibroma, and vascular lesions. Malignant lesions represented by non-melanoma skin cancer, cutaneous lymphoma, metastatic carcinoma, and sarcoma should also be considered differential diagnoses.^15^

Histopathologically, the diagnosis can also be challenging, as MCC is similar to a variety of other tumors that have small, rounded blue cells, including lymphomas, amelanotic melanoma, cutaneous metastases from small cell lung carcinoma,^3^ and other metastatic carcinomas (neuroblastoma, rhabdomyosarcoma, desmoplastic small cell tumors, mesenchymal chondrosarcoma, Ewing's sarcoma, and osteosarcoma).^8^ MCC *in situ*, in which neoplastic cells are limited to the epidermis and/or follicular epithelium, may be mistaken for squamous cell carcinoma *in situ*, melanoma *in situ*, or other pagetoid intraepidermal neoplasia.^20^

Due to similar morphological features, the most challenging differential diagnosis is metastatic small-cell lung cancer,^3^, ^8^ which requires a panel of immunohistochemical markers to define the diagnosis. CK20 and thyroid transcription factor 1 (TTF-1) have increased sensitivity and specificity for excluding small cell lung cancer,^8^ with CK20 being positive in 70%–100% of MCC cases and negative in small cell lung cancer, whereas TTF-1 is always negative in MCC and positive in more than 80% of small cell lung cancer cases.^3^, ^8^, ^14^

## Staging

The current 8^th^ edition of the American Joint Committee on Cancer (AJCC) staging system is based on an updated analysis of 9387 cases of MCC from the National Cancer Database, with a median follow-up of 28.2 months (Table 1).^24^ According to the AJCC, the following parameters are required for the staging of MCC: 1) Maximum clinically measured tumor diameter, prior to resection, and 2) tumor extent (fascia, muscle, cartilage, or bone invasion).^24^ Tumor diameter has prognostic value, being significantly associated with lymph node involvement, disease-specific survival (DSS), and OS.^14^

**Table 1:** Staging of Merkel cell carcinoma – AJCC 8^th^ edition.

Stage	Primary Tumor	Lymph nodes	Metastases
0	*In situ* (restricted to the epidermis)	No metastasis in regional lymph nodes	No distant metastasis
I	Maximum clinical tumor diameter ≤2 cm	cN0, no metastasis to regional lymph nodes on clinical or radiological evaluation	No distant metastasis
		pN0, no metastasis to regional lymph nodes detected in the histopathological evaluation	
IIA	Maximum clinical tumor diameter >2 cm	cN0, no metastasis to regional lymph nodes on clinical or radiological evaluation	No distant metastasis
		pN0, no metastasis to regional lymph nodes detected in histopathological evaluation	
IIB	Primary tumor invades fascia, muscle, cartilage, or bone	cN0, no metastasis to regional lymph nodes on clinical or radiological evaluation	No distant metastasis
		pN0, no metastasis to regional lymph nodes detected in histopathological evaluation	
III (clinical)	Tumor of any size, including invasive tumors and unknown primary tumor	Clinically detected regional lymph node metastasis, in-transit metastasis^a^ with or without lymph node metastasis	No distant metastasis
IIIA (pathological)	Tumor of any size, including invasive tumors and unknown primary tumor	Clinically occult lymph node metastasis identified only by SLNB or lymph node dissection	No distant metastasis
		Clinically or radiologically detected regional lymph node metastasis, pathologically confirmed	
IIIB (pathological)	Tumor of any size, including invasive tumors	Clinically or radiologically detected regional lymph node metastasis, pathologically confirmed, in-transit metastasis^a^ with or without lymph node metastasis	
IV	Tumor of any size, including invasive tumors and unknown primary tumor	Any N	Metastasis beyond regional lymph nodes

a In-transit metastasis: a tumor distinct from the primary lesion and located between the primary lesion and regional lymph nodes or distal to the primary lesion.

MCC is characteristically an aggressive, locally invasive tumor, with a high incidence of local recurrence and regional lymph node involvement.^1^ The SLNB is an important staging tool recommended for all patients with a clinically negative node, to perform pathological node staging whenever possible.^14^

According to the 8th edition of the AJCC staging system, the five-year OS estimates for the clinical and pathological staging of local disease were 45.0% and 62.8% for stage I; 30.9% and 54.6% for stage IIA and 27.3% and 34.8% for stage IIB, respectively. The five-year OS for revised stage IIIA was 40.3%, while it was 26.8%.for stage IIIB.^24^ In stage IV disease, the two-year survival rate is only 26%.^1^

Sihto et al., in 2009, reported that polyomavirus-positive MCC had a higher survival rate when compared to polyomavirus-negative tumors, with a five-year survival rate of 45% *vs*. 13% (p-value < 0.01), respectively.^12^

## Treatment

### Surgery for the primary tumor

Surgery is the initial treatment modality for most cases of MCC. The definition of surgical margins is still a controversial topic.^14^ The goal of surgical treatment is a negative histological margin, and in general, surgical excision with margins of 1–2 cm remains the first step in tumor management.^25^ Boyer et al. analyzed 45 patients with stage I MCC and reported a median margin of 16.7 mm of clinically normal skin, necessary for the absence of histopathological involvement.^26^

A study of 1795 patients with stage I and II MCC showed no differences between wide surgical excision and Mohs micrographic surgery regarding the presence of residual tumor at the surgical margins, and no difference was observed in OS between the two treatment modalities.^27^ Histopathologically negative margins are related to better local control and survival, especially in cases of localized disease treated solely with surgery. In all cases of surgical treatment, SLNB should be planned before a definitive excision, as the surgery may alter lymphatic drainage.^14^

Santamaria-Barria et al. elucidated the role of SLNB in the management of MCC in a review of 161 patients, identifying micrometastases in one-third of them.^28^ Thus, after checking for free margins and, if indicated, SLNB, the challenge is to determine whether or not to offer adjuvant treatment.

### Radiotherapy

Radiotherapy (RT) plays an important role in the treatment of MCC. MCC is a radiosensitive neoplasm and RT may be considered as primary therapy in patients who are not candidates for surgery.^29^, ^30^ Although RT alone is inferior to surgical resection because of the risk of distant MCC recurrence, it can be used when surgery is contraindicated,^31^, ^32^, ^33^ with good locoregional control and five-year OS of up to 40%–60% in patients with primary macroscopic and/or nodal metastases.^33^

Data are conflicting regarding its use as adjuvant therapy. Postoperative adjuvant RT seems to increase local tumor control but has no significant impact on tumor-related overall survival.^34^, ^35^ In a review of head and neck MCC cases only, adjuvant RT showed significant benefit in survival over surgery alone, and chemotherapy (CT) plus RT offered an advantage over RT alone in cases of large tumors (>3 cm) or positive margins.^36^ A retrospective analysis of stage I to III MCC showed a low recurrence rate (3%) in patients with clinically-negative nodes treated with appropriate surgery (including SLNB) and the selective use of adjuvant RT for high-risk tumors, including lymphovascular invasion; second malignancy (leukemia/lymphoma) at diagnosis, multiple positive lymph nodes, and extracapsular extension; tumors >2 cm. The authors also conclude that, with adequate surgery, the routine use of adjunctive local RT is unlikely to be beneficial for the vast majority of patients.^37^ Moreover, some data have suggested that adjuvant RT may be omitted for those with small primary tumors (<1 cm), free surgical margins, negative SLNB, and the absence of poor prognostic factors, such as lymphovascular invasion and immunodeficiency.^38^

A retrospective analysis of 46 low-risk head and neck MCC cases treated between 2006 and 2015 demonstrated that failure to perform postoperative RT was associated with a significantly higher risk of local recurrence (five-year local recurrence of 26% in the group treated without adjuvant RT *versus* 0% in the group that received adjuvant RT, p = 0.02). Patients were considered low-risk if they had a primary tumor ≤ 2.0 cm in diameter, microscopically negative margins on surgical excision, negative SLNB, and no chronic immunosuppression.^39^

Jouary Y et al. showed a significant reduction in local recurrence with no improvement in overall survival with adjuvant RT in a study conducted before the introduction of SLNB in the treatment of MCC.^40^

In a retrospective non-randomized cohort of 6908 cases from the US National Cancer Database, adjuvant RT was associated with improved survival in patients with localized MCC (stages I and II) compared with patients who underwent surgical treatment alone (stage I: Hazard Ratio [HR = 0.71], 95% CI: 0.64‒0.80, p < 0.001; stage II: HR = 0.77, 95% CI: 0.66‒0.89, p < 0.001). However, in patients with nodal involvement (stage III), there was no statistically significant difference in OS between patients who had surgery followed by RT compared to those submitted to surgery alone (HR = 0.98, 95% CI: 0.86–1.12, p = 0.80).^41^

In contrast, a retrospective study from the Moffitt Cancer Center, evaluating 171 patients, found an improvement in locoregional control and OS in patients with pathologically or clinically positive lymph nodes treated with postoperative RT. In this publication, the patients were treated with wide local excision (1–2 cm margins) and all of them underwent nodal staging.^42^

Thus, the NCCN included RT as an adjunctive treatment option for all MCC stages and should ideally be performed within 4–6 weeks of surgery, as the delay has been associated with worse outcomes.^14^

## Systemic treatment

### Chemotherapy

The role of adjuvant CT for MCC is less well defined. Hasan et al. performed a large systematic review and found that adjuvant RT resulted in significantly higher three-year local control rates, decreased recurrence rates, and improved three-year OS rates, whereas adjuvant CT provided no benefits.^43^ Moreover, additional studies suggest that the effect of adjuvant chemotherapy on recurrence is unclear or that there is no significant survival improvement when compared to adjuvant RT alone.^29^, ^44^, ^45^

For many years, CT was the only option available as treatment for advanced disease. MCC is considered a chemosensitive tumor and treatment regimens of platinum with etoposide or taxanes and anthracyclines alone or in combination are initially effective, with a response rate of up to 75%, but usually, the duration of response is short, not resulting in survival benefit. Median progression-free survival (PFS) ranges from three to eight months, and the duration of partial response is only three months for the first-line therapy.^25^, ^46^ Given the advanced age of many patients and their comorbidities, CT toxicity can severely affect patients quality of life.

### Immunotherapy

Immunotherapies are currently the center of attention, since the importance of the immune system in MCC control has been described, with PD-1 (programmed cell death 1) and PD-L1 (programmed cell death protein ligand 1) inhibitors being promising options in cases of metastatic disease.^21^

The PD-1 and PD-L1/PD-L2 pathways constitute a complex system of receptors and ligands involved in controlling T-cell activation. In most tumors, PD-L1 is predominantly expressed in tumor cells and PD-1 in lymphocytes that infiltrate the tumor.^1^ However, PD-1 can be expressed not only by CD8+ T lymphocytes, but also by CD4+, CD20+, Treg, and NK cells.^47^ The binding between PD-1 and its PD-L1 ligand leads to the inactivation and decreased proliferation of T cells. This process seems to be an important mechanism for the immune response inhibition by the tumor.^48^

The association of immunosuppression with increased risk of MCC, in addition to some data that show a better prognosis in tumors with high infiltration of CD8+ T lymphocytes, has become a justification for immunotherapy use.^15^, ^49^ Additionally, Lipson et al., in 2013, reported a significant association between the presence of MCPyV and PD-L1 expression in the tumor and between the presence of MCPyV and a moderate to intense inflammatory infiltrate. These findings suggest that an immune response to viral antigens creates a local pro-inflammatory environment that stimulates PD-L1 expression in the tumor.^50^ These findings paved the way for the use of immune checkpoint inhibitors in metastatic MCC.

The first study in this scenario was JAVELIN Merkel 200, an open, phase II multicenter clinical trial that investigated the clinical activity and safety of avelumab, an anti-PD-L1 antibody. The study features part A, performed in patients with MCC who were previously exposed to CT,^51^ and part B, performed in patients who had not received prior systemic treatment for metastatic disease.^52^ In part A, 88 patients were treated with avelumab and the confirmed overall response rate (ORR) was 33% (including ten complete responses) with 26% of patients without disease progression at two years. The median duration of the response was not reached and the median OS was 12.6 months.^51^ An objective response of 34.5% was found in PD-L1 positive *versus* only 18.8% in PDL-1 negative patients (expression <1%). Considering the 5% cutoff in PD-L1 expression; 52.6% of PD-L1 positive patients *versus* 23.6% of PD-L1 negative patients had an objective response. Furthermore; 26.1% of patients with MCPyV-positive tumors compared to 35.5% with MCPyV-negative tumors had a response.^53^ Another subgroup analysis suggested a greater likelihood of response in patients who received fewer prior systemic therapies, which was confirmed in part B of the Javelin Merkel 200 trial. The objective response among the 29 patients included in the first-line scenario was 62%, with 83% of responses lasting six months or more.^52^

Munhoz et al., in 2020, described the experience with avelumab as a second-line treatment (or first-line in patients not candidates for chemotherapy) in a subset of 46 Latin American patients (mean age: 71.6 years; 60.9% male; mean duration of treatment: 7.9 months). Objective responses were observed in 19 patients (objective response: 57.9%; complete response: 15.8%; partial response: 42.1% and stable disease: 10.5%) and safety was consistent with global data.^54^

Another study with similar characteristics demonstrated the effectiveness of pembrolizumab in MCC, an anti-PD-1 monoclonal antibody. A total of 50 patients with advanced MCC were treated with an objective response of 56% and a six-month PFS rate of 67%. In contrast to the avelumab study, a higher response rate was seen in MCPyV-positive tumors (62%) than in MCPyV-negative ones (44%).^55^

In the CheckMate 358 study, nivolumab, another anti-PD-1, was evaluated in 25 patients with advanced MCC, with an objective response of 64%. The responses occurred in treatment-naïve patients (71%) as well as those with at least one prior systemic therapy (63%), with a short median time to response (75% of patients responded within two months).^56^

The summary of recent studies with immunotherapy for the treatment of metastatic MCC is depicted in Table 2.^52^, ^56^, ^57^, ^58^

**Table 2:** Important data from recent clinical trials involving immunotherapy^52^, ^56^, ^57^, ^58^.

	Study name	Drug class	Type of study	Number of patients	ORR (%)	PFS at 24 m (%)	OS at 24 m (%)	References
Avelumab	Javelin 200 ‒ Part A	Anti-PD-L1	2^nd^ line or subsequent lines	88	33	26	36	Kaufman 2018^57^
Avelumab	Javelin 200 ‒ Part B	Anti-PD-L1	1^st^ line	29	62	NR	NR	D’Angelo, 2018^52^
Pembrolizumab	Keynote ‒ 017	Anti-PD-1	1^st^ line	50	56	48.3	68.7	Nghiem, 2019^58^
Nivolumab	CheckMate 358	Anti-PD-1	1^st^ line	15	71	NR	NR	Topalian, 2017^56^
			2^nd^ line	10	63			

ORR, Overall response rate; PFS, Progression free survival; OS, Overall Survival; NR, Not reported.

Given the efficacy of immunotherapy for metastatic MCC, the next challenge is to evaluate such treatments in the adjuvant scenario. Due to the high risk of MCC recurrence, despite the initial treatment and lack of benefit from cytotoxic treatment, its investigation is highly relevant, covering an unmet medical need. Two-phase II trials with nivolumab and avelumab are evaluating the use of immune checkpoint inhibitors for high-risk patients yet without officially published results. The neoadjuvant scenario with the CheckMate 358 trial, involving 39 patients with stage IIA to IV resectable MCC, evaluated the use of nivolumab (≥1 dose) approximately four weeks before surgery. Three patients were not submitted to surgery due to tumor progression and 36 patients were submitted, of which 17 (42.2%) attained a complete pathological response. Among 33 radiographically evaluated patients who underwent surgery, 18 (54.5%) showed tumor reduction ≥30%. No patient with a pathological complete response had tumor recurrence during the observation period.^59^

## Conclusion

MCC is a rare and aggressive disease, whose incidence is increasing. In recent decades, many advances have been made regarding the knowledge related to the biology, diagnosis and treatment of MCC, but great challenges still remain. Primary tumors should be treated with complete surgical excision with adequate surgical margins. Adjuvant RT should be considered. Regarding advanced MCC, immunotherapy has drastically changed the standard of care, as it is superior to chemotherapy. Remarkable responses have been seen in MCC patients treated with PD-1/PD-L1 axis inhibitors and it seems to be a promising way to change the prognosis of this aggressive skin cancer.

## Financial support

None declared.

## Authors' contributions

Stella Meireles Siqueira: Statistical analysis; drafting and editing of the manuscript; collection, analysis, and interpretation of data; intellectual participation in propaedeutic and/or therapeutic conduct of studied cases; critical review of the literature.

Gabriella Campos-do-Carmo: Statistical analysis; drafting and editing of the manuscript; collection, analysis, and interpretation of data; intellectual participation in the propaedeutic and/or therapeutic conduct of the studied cases.

Alexssandra Lima Siqueira dos Santos: Statistical analysis; drafting and editing of the manuscript; collection, analysis, and interpretation of data; intellectual participation in the propaedeutic and/or therapeutic conduct of the studied cases; critical review of the literature.

Cícero Martins: Statistical analysis; drafting and editing of the manuscript; collection, analysis, and interpretation of data; intellectual participation in the propaedeutic and/or therapeutic conduct of the studied cases; critical review of the literature.

Andreia Cristina de Melo: Statistical analysis; approval of the final version of the manuscript; design and planning of the study; drafting and editing of the manuscript; collection, analysis, and interpretation of data; effective participation in research orientation; intellectual participation in the propaedeutic and/or therapeutic conduct of the studied cases; critical review of the manuscript.

## Conflicts of interest

None declared.
